# Proceedings of the American Medical Association

**Published:** 1858-08

**Authors:** 


### *Proceedings of the American Medical Association.*

Continued from June No.

Dr. Hamilton, from the committee of delegates from medical schools and colleges, to whom was referred the report of the special committee on medical colleges, reported the following preamble and resolution :

Fully appreciating the value and importance of the resolution under which they were appointed, but a majority of the gentlemen constituting this committee not being authorized by the medical faculties of the several colleges with which we are connected, to act as their representatives in this matter, and therefore regarding it quite impossible to secure a convention of delegates in the interim of the meetings of the association: therefore—

*Resoolved*, that we recommend to all the medical colleges entitled to a representation in this body, that they appoint delegates, especially instructed to represent them in a meeting to be held at Louisville, on Monday, the day immediately preceding the convention of the American Medical Association, for the year 1859, at 10 o'clock, at such place as the committee of arrangements shall designate.

The report was accepted, and the preamble and resolution were passed; after which several gentlemen claimed the floor, but the President decided that the reports of special committees were in order, the reports of committees on medical topography and epidemics having previously been referred to the committee on publication, without reading.

Dr. S. M. Bemis, of Kentucky, read an able and learned report on the "influence of marriages of consanguinity upon offspring."

Dr. John L. Atlee, from the committee appointed at the annual meeting at Richmond, in May, 1852, to procure a stone, with a suitable inscription, to be inserted in the Washington National Monument, made a final report. It stated that Mr. Haldy, a marble mason in the city of Lancaster, Pa., had in his employment a young man, Mr. J. Augustus Beck, a native of Litiz, Pa., who had given unmistakable evidence of genius as a sculptor. At the suggestion of the late lamented Dr. A. L. Pierson, of Salem, Mass., (made at the meeting in New York, just two days before his death,) the design of the celebrated painting of Girodet-Tricoson, representing Hippocrates refusing the presents of the Persian King, Artaxerxes, and his invitation to leave Greece, and reside and practice among her enemies, was selected. This was sculptured upon a block of Vermont marble, with the motto—"*Vincet Amor Patriæ*," and the stone is at the monument grounds. The entire expense was $1,000, of which one-half was paid to the young artist. The amount contributed by members individually was $501 30; the balance was voted from the treasury of the society. Accompanying the report was a letter from the Secretary of the Washington National Monument Association, and a resolution of thanks to the railroad companies, by whose liberality the stone was brought from Lancaster to Washington free of charge. The report was accepted, and the resolution was passed.

Dr. Palmer, of Buffalo, read a report made by Dr. E. Andrews, of Chicago, Illinois, on the "functions of different portions of the cerebellum," of which the following is an abbreviated report:

The cerebellum is divided into three lobes, one median and two lateral.

The muscular system of most animals is divided into two groups, viz : those which act upon the anterior extremities and the adjacent parts of the trunk, and those which move the posterior extremities and the corresponding portion of the trunk.

The report shows that there is a direct ratio between the strength and bulk of the anterior group of muscles and the size of the median lobe of the cerebellum.

Also that the lateral lobes manifest a double ratio, their size being as the strength of the posterior group of muscles, and also the size of the hemisphere of the cerebrum.

It is inferred that the action of the cerebellum is to *excite* motion, and not merely to *co-ordinate* it; that the influence of the median lobe is chiefly expended upon the anterior group of muscles, and that the action of the lateral lobes is in some manner double, part of their influence acting upon the posterior group of muscles, and part of it having some relation to mental power, whose exact nature is not yet understood.

The facts and arguments are drawn from comparative anatomy, and illustrated with outline drawings.

Dr. Campbell, of Georgia, read a report on the "nervous concomitants of febrile diseases," which was accepted and referred to the committee of publication.

Dr. J. Marion Simms, of New York city, read an abstract of his report on the treatment of results of obstructed labor, illustrated with a series of illustrations. The Doctor seemed perfectly familiar with his subject, and was frequently applauded.

Dr. Stephenson, of New York, read an interesting abstract of his report on "the treatment best adapted to each variety of cataract, with the method of operation, place of selection, time, age, &c." Dr. Stephenson is the Surgical Director of the New York Opthalmic Hopital, an institution which relieves hundreds every year from sufferings with diseases of the eye.

On motion, other reports were called for, read by their titles, and referred to the committee of publication.

Dr. Edwards, from the committee of nomination, offered the following list of committees for the ensuing year, which was accepted, and the committees were chosen :

*Special Committee on the Microscope.*—Drs. Holsten of Ohio, Dalton of New York, Hutchinson of Indiana, Stout of California, and Ellis of Massachusetts.

*Special Committee on Medical Jurisprudence.*—Drs. Smith of New York, Hamilton of Buffalo, Crosby of New Hampshire, Purple of N. York, and Mulford of New Jersey.

*Committee on Quarantine.*—Dr.s Harris of New York, Moriarty of Massachusetts, La Roche of Pennsylvania, Wragg of South Carolina, and Fenner of New Orleans.

*Committee on Surgical Pathology.*—Dr. James R. Wood, of New York.

*Committee on Diseases and Mortality of Boarding Schools.*—Dr. C. P. Mattingly, of Kentucky.

*Committee on the various Surgical Operations for the relief of Defective Vision.*—Dr. Montrose A. Pallen, of St. Louis.

*Committee on Milk Sickness.*—Dr. Edward A. Murphy, of Indiana.

*Committee on Medical Ethics.*—Drs. John Watson of New York, Dalton of Massachusetts, Emerson of Pennsylvania, Hamilton of New York, and Gaillard of South Carolina.

Dr. Edwards also reported from the Committee of nomination the following resolution.

*Resolved,* That a committee of nine be appointed by the chair to wait on the Hon. Howell Cobb, Secretary of the Treasury, and request the restoration of Dr. M. J. Bailey, as inspector of drugs and medicines for the port of New York.

After a long disccussion the resolution, amended as follows, was then carried by a vote of 79 ayes to 55 noes.

*Resolved,* That a Committee of nine be appointed by the chair to wait on the Hon. Howell Cobb, Secretary of the Treasury, and respectfully to request the restoration of Dr. M. J. Bailey as inspector of drugs and medicines for the port of New York—at the same time disclaiming all political considerations.

Dr. Bohrer, of Georgetown, chairman of the committee on special medical essays, stated that they had not had time to read, much less to consider, the papers placed in their hands.

On motion, the committee on special medical essays was instructed to hand such papers as they deemed worthy to the committee on publication.

The President announced, as a special committee to wait on the Secretary of the Treasury, Drs. Arnold of Georgia, Atkinson of Virginia, Buckley of New York, Hayes of Pennsylvania, Smith of New Jersey, McPheeters of Missouri, Hargraves of Alabama, Pitcher of Michigan, and Hooker of New York.

On motion, Dr. Edwards was added to the committee as chairman. He declined, giving personal reasons as an excuse, but the Convention refused to receive it, and he was accordingly chosen.

A gentleman stated that he, with several friends, had voted for the resolution with the sole intention of moving its reconsideration.

Dr. Grant of New Jersey, presented a complaint made by the Newark Medical Society against the Medical College of New York, for a violation of the ethics of the profession. Dr. Edwards presented a similar complaint, and Dr. Oakley a complaint from the Union and Essex county medical societies. They were received and referred.

Dr. Sutton, of Kentucky, moved that Dr. Jarvis, of Massachusetts, have further time to report on a uniform system of registration of births, marriages and deaths, and that a committee be appointed to urge upon the census bureau of 1860 the importance of having a physician attached to it to collect vital statistics.

Dr. Kyle, of Ohio, proposed an amendment to the constitution by which no person can sit as a member or a delegate at meetings of this association who is not a graduate of a recognized medical college. Laid over for one year, under the rules.

Dr. L. A. Smith presented resolutions of the New Jersey Medical Society, praying for such changes in the constitution as would establish a board of census in every judicial circuit of the Supreme Court, who should examine and grant diplomas to all proper members of this association. Laid over for one year, under the rules.

Dr. Humphries, of Indiana presented a resolution praying for an interchange of transactions of State and County Societies; which was adopted.

Dr. Boyle, chairman of the committee of arrangements, presented the names of Professor Swallow, of Missouri, and Professor Mittag, as "members by invitation," and they were elected,

An invitation from Professor Bache to visit the Coast Survey bureaux on Capitol hill, was read, and a vote of thanks for the courtesy was passed.

Dr. Gibbs, of South Carolina, moved that Prof, Henry be requested to favor the association with his views on meteorology at such time during the session as he may select; carried unanimeusly.

Dr. Campbell, of Georgia, moved that the Secretary place on record an expression of regret with which the society has learned the death of Drs. C. R. Walton, S. W. Clanton, Marshall Hall, T. Y. Simmons, J. R. Mitchell, and other members deceased since the last annual session; carried.

On motion of Dr. Phelps, the following resolutions were passed unanimously, the members rising:

*Resolved,* That the thanks of this association are eminently due to the Regents and Professor Henry, of the Smithsonian Institution, for the ample and convenient accommodation afforded for the transaction of business·

*Resolved,* That the Committee of arrangements are entitled to our praise and highest appreciation of their exertions to promote the comfort of the members and the best interest of the association.

*Resolved,* That to the Physicians of Washington and Georgetown, and the Faculty of Georgetown College we accord the homage of our sincerest thanks for their elegant hospitalities extended to the members from abroad, by which the pleasure of their sojourn here has been so greatly enhanced.

*Resolved,* That we feel assured that the impression on the tablet of memory received here, in our national metropolis, in this the first year of the second decade of the association, will long remain an evidence of the urbane attentions received not only from the Chief Magistrate and other public functionaries of our glorious Union, but of private citizens and the community at large.

*Resolved,* That the manifestations of union of heart and purpose in the action of this session, inaugurate a new era, and call for devout acknowledgment to Divine Providence, and presage, as we trust, not only a bright future for the association, but also contributing to the perpetuity and prosperity of our great national confederation.

On motion of Dr. Anderson of New Jersey, it was unanimously resolved that the thanks of the medical association be presented to Rev. Dr. McGuire and the faculty of the College of Georgetown for their very cordial reception and entertainment of the association at the College yesterday.

On motion of Dr. Foster, of Tennessee, it was resolved that after 1860 Dr. Hamilton have the privilege of using his report on "deformities after fracture," published in the Transactions, for a work which he proposes to publish.

Dr. Duhamel, of Washington, moved that a committee be appointed to investigate and report upon the "National Hotel disease.",

Dr. Foster, of Tennessee, opposed the appointment of such a committee, as did Dr. Boyle, of Washington. Dr. Duhamel withdrew his motion.

Dr. Gaston, of South Carolina, exhibited a new uterine supporter of novel construction.

Dr. Campbell, of Georgia, was not aware, until he had just heard permission granted to Dr. Hamilton, that he had transgressed in republishing in a work a report which he had contributed to the transactions of the association. [Cries of "Regret it," "regret it."] He did regret it, and asked the sanction of the society; which was granted.

The president appointed Drs. Miller, Antisel, and Garnet a committee to wait on the census bureau, as provided by the resolution of Dr. Sutton.

Dr. Dunbar moved a reconsideration of the vote appointing a committee to request the reinstation of Dr. Bailey, and Dr. Morgan seconded it, but, as Dr. Parker had been invited upon the platform, the motion was ruled out of order.

*Dr. Parker's Chinese Hospital.*

Dr. Peter Parker, ex-commissioner to China, was then introduced and received with applause. He exhibited some curious specimens of *calculi*, as the results of thirty-eight operations upon Chinese. They were of various shapes and composition, and weighed from a few drachms up to three, seven and eight ounces. His description of the operation by which these calculi were removed, was deeply interesting, and it was gratifying to learn that out of the thirty-eight patients all but five or six recovered perfect health.

Dr. P. proceeded to state that he has treated in China at the Hospital under his charge, fifty-three thousand cases. Pictures of the most curious cases he had brought to this country, and they were on exhibition in the room below. At no very distant period he hopes to place in a permanent form the result of his labors, with illustrations. Among other cases he had probably performed upwards of a thousand operations for cataract. On one day he operated in sixteen cases, the youngest being a mere child, and the oldest an old lady seventy-nine years of age. She came, led by a servant, submitted heroically to operations on both eyes the same day, and in a fortnight had her sight perfectly restored. In acknowledging a vote of thanks, Dr. Parker said he had among his patients all classes, from members of the imperial family down to beggars. His greatest difficulty had been to persuade his patients that he could not cure all diseases.

Dr. Dunbar claimed the floor, and urged the reconsideration of the vote appointing a committee to wait on the Secretary of the Treasury, and solicit the reinstation of Dr. Bailey.

Dr. Paine, of Virginia, opposed the reconsideration.

Dr. Tyler advocated it, and asked if this association was formed to wait on executive officers, and to dictate to them who they shall remove and who they shall appoint. Many gentlemen around him, he was assured, had voted for the resolution without due reflection, and he trusted with confidence in their sober second thought- The press and the profession, he felt confident, would denounce this association if it entered into the wide field of politics. It was instituted to promote the great cause of science, not to join issue with government.

Dr. Morgan also advocated a reconsideration. He was not a partisan. Although he resides in Washington, he has no personal acquaintance with the President or the Secretary of the Treasury, but he was confident they would not have made the change without good reason, and it was not the mission of this association to criticise or attempt to change their views.

Dr. Palmer, after stating how little regard he had for the opinions of the press, inquired as to the present incumbent of the office. Is he capable?

Dr. Watson, of New York, said that Dr. Bailey had had his circulars out since his "rotation," and the subject had been twice before the Academy of Medicine, who have ignored it.

Dr. Burns, of Brooklyn, said that he was not a politician, and that he was a personal friend of Dr. Bailey, but he hoped that the vote would be reconsidered.

A member from California related his experience there on a question as to the superintendent of a lunatic asylum. In his opinion the less the association had to do with politics, or with expressions of opinion on political appointment, the better.

Dr. McNulty, of New York, said that the question had been twice before the New York Academy of Medicine, and twice been voted down. The present incumbent, whom it is sought to oust, is a German by birth and education. He can read the invoices in whatever European language they may be sent, and he makes his own analyses, which it is reported the ex-inspector did not do.

After some "parliamentary" skirmishing, it was decided to reconsider by a vote of 51 ayes to 32 noes. And, on motion, the subject was indefinitely postponed.

The Association then took a recess of two hours, for dinner.

*Evening and closing Session.*

The Association was called to order at 5 o'clock P. M., by Dr. Sutton, one of the Vice Presidents, who took the chair.

The amendments to the constitution, proposed at the annual meeting in Nashville, had been made the special order. They were

1st. Amend the third article of the Constitution, in relation to meetings, by inserting after the words "first Tuesday in May," the words "or the first Tuesday in June;" and also inserting after the words "shall be determined," the words "with the time of meeting."

2d. In article 2, omit the words "medical colleges," and also the words "the faculty of every regular constituted medical college, or chartered school of medicine, shall have the privilege of sending two delegates."

Each amendment was separately discussed, and each was lost by a large vote. An amendment proposed at Philadelphia in 1856 providing for the establishment of a permanent secretaryship was lost by a vote of 53 ayes to 84 noes.

On motion of Dr. Foster, of Tennessee, the secretary was directed to collect all the by-laws, and have them printed in the next volume.

An attempt was made to introduce a motion endorsing the accoustics and ventilation of the new Capitol extensions, but it was ridiculed by Dr. Sayre, and was withdrawn.

Various additional votes of thanks were passed, aud, at ten minutes of seven, the association adjourned *sine die.*

